# High Mobility Group A Proteins as Tumor Markers

**DOI:** 10.3389/fmed.2015.00015

**Published:** 2015-03-25

**Authors:** Pierlorenzo Pallante, Romina Sepe, Francesca Puca, Alfredo Fusco

**Affiliations:** ^1^Istituto per l’Endocrinologia e l’Oncologia Sperimentale, Consiglio Nazionale delle Ricerche, Dipartimento di Medicina Molecolare e Biotecnologie Mediche, Università degli Studi di Napoli Federico II, Naples, Italy; ^2^Instituto Nacional de Câncer, Rio de Janeiro, Brazil

**Keywords:** HMGA1, HMGA2, cancer marker, diagnosis, prognosis

## Abstract

Almost 30 years ago, overexpression of HMGA proteins was associated with malignant phenotype of rat thyroid cells transformed with murine retroviruses. Thereafter, several studies have analyzed HMGA expression in a wide range of human neoplasias. Here, we summarize all these results that, in the large majority of the cases, confirm the association of HMGA overexpression with high malignant phenotype as outlined by chemoresistance, spreading of metastases, and a global poor survival. Even though HMGA proteins’ overexpression indicates a poor prognosis in almost all malignancies, their detection may be particularly useful in determining the prognosis of breast, lung, and colon carcinomas, suggesting for the treatment a more aggressive therapy. In particular, the expression of HMGA2 in lung carcinomas is frequently associated with the presence of metastases. Moreover, recent data revealed that often the cause for the high HMGA proteins levels detected in human malignancies is a deregulated expression of non-coding RNA. Therefore, the HMGA proteins represent tumor markers whose detection can be a valid tool for the diagnosis and prognosis of neoplastic diseases.

## Tumor Markers

In presence of an evident tumor mass in the organism, cancer cells tend to produce high levels of particular substances collectively named tumor markers. Tumor markers are generally found in body fluids (including blood serum and urine) and tissues of several cancer patients and are mainly represented by protein macromolecules (1). However, the use of such markers in clinical diagnostic shows some critical aspects. Indeed, conditions not related to the presence of tumor could increase their levels, while patients affected by cancer could not display an increase of their tumor markers, due to a specific intrinsic variability. Tumor markers could be associated with a particular type of cancer or with multiple cancers, but no marker has been virtually associated with any specific cancer type so far. Nevertheless, in some instances, markers are very useful in clinical practice, and currently about 20 markers have been characterized and are used (1), but still for several kind of cancers, no marker is available.

To correctly make pathological report, the evaluation of tumor marker needs to be completed with other kind of analysis, like assessment of bioptic tumoral tissue. Moreover, during the administration of therapeutic regimen, evaluation of particular tumor markers could be used to check the patients’ response, while after treatment, they can be used to monitor recurrence of cancer (1). However, the aim of current and future studies is the detection of tumor markers before the treatment is starting, allowing the most appropriate choice for anti-cancer therapy.

About 30 years ago, the high mobility group A (HMGA) proteins were suggested to represent a powerful tumor marker in relation to malignancies. Indeed, the analysis of chromatin-related proteins in rat thyroid cells transformed by acute murine retroviruses revealed an abundant expression of these proteins only in fully transformed thyroid cells able to grow in semisolid media and to induce formation of tumors in nude mice (2, 3). Conversely, these proteins were not expressed at all in uninfected cells or retrovirally infected ones, that did not show the above mentioned growth characteristics, despite having lost their thyroid differentiation markers (3).

HMGA proteins are encoded by two genes, *HMGA1* and *HMGA2*, located at chromosome 6p21 and 12q13–15, respectively. *HMGA1* gene generates two proteins, HMGA1a and HMGA1b, by alternative splicing. The three proteins of the HMGA family (HMGA1a, HMGA1b, and HMGA2) share an analogous structure (107, 96, and 108 amino acids, respectively), being well preserved alongside the evolution (4, 5). They contain three AT-hook basic domains conferring them the ability to bind the DNA minor groove at sequences rich of A and T nucleotides, and to assemble transcriptional or enhancer complexes on chromatin (6). An additional COOH-terminal domain still maintains an uncharacterized activity. Specifically, HMGA1a contains additional 11 amino acids between the first two AT-hook domains if compared with HMGA1b, and both are lacking the COOH-domain that, conversely, is present in HMGA2 (7). In addition, HMGA1a and HMGA1b are different from HMGA2 in at least two amino acidic stretches of 25 and 12 residues, respectively.

As far as the HMGA expression is concerned, these two genes are both abundantly expressed in embryonic phases, whereas they are present at very low levels in adult tissues (8, 9). In particular, HMGA2 is only, very weakly, expressed in preadipocytic proliferating cells (10), spermatids, and spermatocytes (11, 12). Conversely, they have been found abundantly overexpressed in human malignancies that suggested causal role for carcinogenesis and tumor progression, as pointed out by the generation and characterization of several experimental models all showing for HMGA a powerful transforming ability (13–16).

Their crucial role in the diverse phases of development has been demonstrated by the generation of *hmga1* and *hmga2* knock-out (ko) mice (17). In particular, *hmga1* ko and heterozygous mice developed cardiac hypertrophy (18) and type 2 diabetes (19), suggesting a role of this protein in the growth of cardiomyocytic cells and in the modulation of insulin pathway. Interestingly, heterozygous and null *hmga2* mice displayed a reduction of body size of 25 and 60% respectively (“pygmy” phenotype) in comparison with the wild-type (wt) mice, suggesting the involvement of hmga2 in the regulation of body size and in the adipocytic differentiation (8, 10). It is worth to note that the concurrent knocking out of both *hmga1* and *hmga2* resulted in a reduction of mouse body size of about 80%, generating the so-called “superpygmy” phenotype (20). The ability of the HMGA proteins to activate the E2F1 transcriptional activity likely accounts for this phenotype (21).

## HMGA and Colon Cancer

The role of HMGA proteins in cancer and, in particular HMGA1, has been widely evaluated in colorectal carcinomas (22, 23) (Table 1).

**Table 1:** **Overexpression of HMGA proteins in several human solid malignancies**.

Type of cancer	HMGA1	HMGA2
Colon and rectum	Abe et al. (22)	Helmke et al. (26)
	Chiappetta et al. (23)	Li et al. (27)
	Cleynen et al. (24)	Wang et al. (28)
	Bush et al. (25)	
Breast	Chiappetta et al. (33)	Wend et al. (35)
	Mansueto et al. (34)	Jones et al. (36)
Pancreas	Piscuoglio et al. (37)	Piscuoglio et al. (37)
	Hristov et al. (38)	
Ovary	Masciullo et al. (39)	Mahajan et al. (40)
		Califano et al. (41)
		Hetland et al. (42)
		Wu et al. (43)
Lung	Zhang et al. (44)	Rice et al. (45)
	Kettunen et al. (46)	Kumar et al. (47)
	Zhang et al. (48)	
Esophagus	Chen et al. (51)	
Testis	Franco et al. (52)	Franco et al. (52)

Several studies reported that HMGA1 was abundantly expressed in colon carcinoma tissue and, conversely, almost undetectable in non-pathological mucosa. Interestingly, the overexpression of HMGA1 was strongly associated with invasive ability, the staining being more intense in invasion-positive cases in comparison to invasion-negative ones (22), in advanced stage (T3 and T4 tumors) and with the presence of distant, but not regional, metastases. It is worth noting that HMGA1 expression (percentage of cells and intensity) increased gradually from pre-malignant stages of colorectal carcinoma to adenoma (characterized by mild to severe atypia) up to carcinoma. Conversely, non-neoplastic polyps did not show HMGA1 overexpression (22, 23). Therefore, these findings seem to indicate that HMGA1 overexpression is associated with early transformation, rather than with colon cell hyperproliferation.

It has been observed that RAS oncogene, activated in a large set of colorectal carcinomas, plays an important role in the modulation of HMGA1 expression. In fact, it is able to induce its expression through the activation of two binding sites responsive to SP1 and AP1 transcription factors (24). In addition, the 5′ region of HMGA1 gene contains also two binding sites for the β-catenin/TCF-4 complex, whose signal transduction activation represents a critical step in colorectal carcinogenesis (25).

As far as the role of HMGA2 in colorectal cancer is concerned, the involvement of this gene is still controversial. Indeed, whereas one study reported that HMGA2 is overexpressed only in 50% of colon carcinoma tissues in comparison to the average expression of normal adjacent mucosa (26), another one showed that HMGA2 expression (evaluated as percentage of stained cells) progressively increased with the severity of carcinoma grade (Dukes’ A–D), in any case, it is absent in non-neoplastic and early adenomas (27). Interestingly, epithelial cells overexpressing HMGA2 resulted located at the invasive front of tissue undergoing epithelial–mesenchymal transition (EMT), and a particular association between HMGA2 overexpression, strong β-catenin staining, and loss of E-cadherin in metastatic lesions was found (27). Finally, it has been also reported that HMGA2 overexpression promotes metastasis formation and affects survival of colorectal cancer patients (28).

Recently, very important advances have been achieved in the identification of mechanisms underlying the development of colon carcinomas. These studies elucidate several pathways involved in the pathogenesis of colonic adenocarcinomas yielding to a subclassification as well as different treatment strategies. Then, it would be very important to correlate the expression of the HMGA proteins with genetic lesions, putting the detection of the HMGA proteins as necessary tool for the appropriate choice of colon cancer therapy.

## HMGA and Breast Cancer

Ongoing studies have analyzed HMGA1 expression in breast carcinomas by using a tissue microarray (TMA) containing more than 1000 carcinoma samples, mainly ductal histotype, complete for the follow-up. HMGA1, not detectable in normal breast tissue, resulted overexpressed in the vast majority of samples analyzed, but no particular association was found with clinico-pathological parameters. Intriguingly, the overexpression of HMGA1 positively correlated with Her2/neu expression and progesterone receptor (PR), while surprisingly, was negatively associated with estrogen receptor (ER). Therefore, these findings suggested for HMGA1 a role in the response to hormonal treatment of particular kind of breast carcinomas. In fact, while it is reported that PR+ breast carcinomas are responsive to hormonal treatment, conversely, ER−/PR+ carcinomas tend to appear in premenopausal and younger patients (29–31) with a worse outcome if compared to younger ER+/PR+ patients (32). Hence, overexpression of HMGA1 could have a prognostic significance based on the endocrine context, probably by influencing the hormonal response and the outcome.

These results confirm previous published data (33) showing that HMGA1 staining was very intense in 40% of hyperplastic lesions characterized by cellular atypia and 60% of ductal carcinomas, whereas the staining was weak in fibroadenomas and in hyperplastic lesions without cellular atypia (Table 1). The same study showed no HMGA1 expression in normal breast tissue (33). These authors also reported that HMGA1 overexpression was comparable between ductal carcinomas of different histological grade, and was associated with c-erbB2 expression (33). It is noteworthy that the analysis of lobular carcinomas, even though performed on a limited number of samples, always showed an intense HMGA1 staining (33, 34).

**Figure 1:**
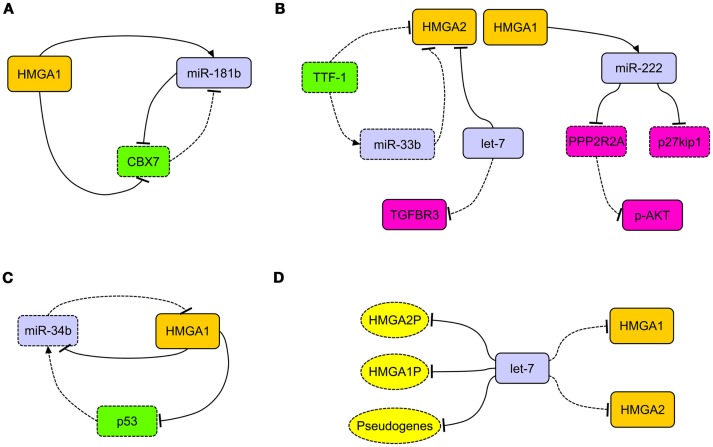
**The network of HMGA regulatory mechanisms in human carcinomas**. **(A)** In breast carcinoma, HMGA1 is able to simultaneously repress the expression of CBX7 and induce the expression of miR-181b. This latter, together with CBX7, takes part to a reciprocal regulation. **(B)** HMGA2, overexpressed in lung carcinomas, acts as competing endogenous RNA for let-7, allowing the activation of the TGF-beta signaling through the upregulation of TGFBR3. Decreased expression of TTF-1 in lung carcinomas allows the overexpression of HMGA2 protein directly, by releasing the transcriptional block on its promoter, and indirectly, by removing the translational block due to miR-33b. HMGA1 is able to induce the expression of miR-222, which in turn can target p27^kip1^ and PPP2R2A, then activating the AKT signaling. **(C)** The loss of miR-34b expression in cancer cells allows the overexpression of HMGA1, which in turn alters the miR-34b pathway by repressing it and its inducer p53. **(D)** The presence of several let-7 binding sites in the 3′ untranslated regions of HMGA1, HMGA2, and relative pseudogenes (HMGA1P, HMGA2P) alters the epigenetic modulation of HMGA2 and HMGA1 themselves (respectively), allowing their overexpression after decoy of let-7 microRNA. Dashed rectangles/ovals and lines represent decreased expression or loss of regulatory action, respectively.

Interestingly, in breast carcinoma, HMGA1 takes part in an important regulatory circuitry involving CBX7 and miR-181b microRNA (miRNA) (34) (Figure 1A). HMGA1 enhances the expression of miR-181b, which in turn, represses the translation of CBX7 mRNA. The transcription of CBX7 is also directly repressed by HMGA1 itself (34) (Figure 1A).

Interesting results were obtained by analyzing HMGA2 expression in breast tumors coming from different geographical areas: 14 samples of breast cancers from African-American patients, 31 samples from Caucasian-American patients, and 14 samples from German patients. A strong nuclear expression of HMGA2 was observed only in the triple negative breast cancers (TNBC), but not in triple positive (TPBC) samples and in “normal” breast tissues adjacent to TNBC samples (35).

HMGA2 has been also detected in phyllodes breast cancers where it was always overexpressed in border line and malignant neoplasias and rarely in benign cases (36), suggesting its involvement during benignity to malignancy transition.

## HMGA and Pancreatic Carcinoma

Several reports indicated that HMGA1 and HMGA2 are abundantly expressed in pancreas adenocarcinomas, where overexpression of HMGA1 correlates with advanced grade and, though less frequently, in pancreas intraepithelial neoplasias (PanIN) (37) (Table 1). Conversely, HMGA1 and HMGA2 were not expressed in normal pancreas. Interestingly, HMGA1 and HMGA2 overexpression correlates with the loss of differentiation and with the presence of lymph node metastases, indicating their involvement in neoplastic transformation and progression (37). The correlation of HMGA overexpression with the acquisition of a more advanced cancer grade is also corroborated by the association found with the poor prognosis of pancreas carcinoma patients, that in general is very short due to high aggressiveness of this type of cancer (37, 38).

Accordingly, all these observations suggest the crucial role played by either HMGA1 and HMGA2 during the progression toward malignancy of pancreatic neoplasias.

## HMGA and Ovarian Carcinoma

HMGA1 was not expressed in normal epithelium surface where adenocarcinomas originated, but it was highly expressed in invasive ovarian carcinomas, and weakly expressed in ovarian carcinomas with low invasive potential (39) (Table 1).

HMGA2 was found to be abundantly overexpressed in papillary serous carcinomas (high grade) and carcinosarcoma (40). Moreover, HMGA2 overexpression correlated with low levels of let-7, a miRNA able to target and repress HMGA2, and with p53 (40). A strong association has been found also with body mass index (BMI) and a combined analysis of these two variables is able to predict the shorter disease-free survival (41). Another study showed that HMGA2 expression did not correlate with the response to chemotherapy and survival, while it was correlated with the expression of several proteins both positively and negatively. Among those with positive correlation, there are Nestin, a cancer stem cell marker, and the gap junction member claudin-7. The negative correlation was found with the mRNA corresponding to the E-cadherin repressor SIP1 (42). The role of HMGA2 in the induction and progression of ovarian cancer has been conclusively demonstrated by a study where HMGA2 was reported to be able to increase proliferation, migration, and metastatic properties of ovarian cancer cells (43).

## HMGA and Lung Cancer

HMGA1 and HMGA2 proteins were overexpressed in non-small cell lung carcinomas (NSCLC), in both squamous and adenocarcinoma histotypes, in comparison with normal lung and benign tissues (44–46) (Table 1). HMGA2 intense nuclear expression was strongly associated with metastases and poor prognosis and, as assessed by Cox multivariate analysis, HMGA2 represents an independent prognostic factor (45). A more recent study confirmed that HMGA2 is highly expressed in metastatic lung adenocarcinoma, where it contributes to cancer progression and metastasis by acting as a competing endogenous RNA for let-7 miRNA family (47). Moreover, the competing action of HMGA2 overexpression is able to activate the TFG-beta signaling by leading to the upregulation of the TGF-beta co-receptor Tgfbr3 (47). Therefore, HMGA2 overexpression would enhance cancer progression, both as a protein-coding gene and as a non-coding RNA (47) (Figure 1B). HMGA1 and HMGA2 may have a role in NSCLC cancer progression also by regulating the expression of miRNAs. Indeed, it has been reported that at least HMGA1 is able to directly regulate the expression of miR-222 in NSCLC cells (48). Since it has been demonstrated that miR-222 can target p27^kip1^, a critical regulator of cell cycle (49), and the phosphatase 2A subunit B (PPP2R2A), which inhibits Akt phosphorylation (48), we can assess that HMGA overexpression contributes to NSCLC progression by dysregulating cell cycle and Akt signaling (48) (Figure 1B).

An important role in the regulation of HMGA2 expression in lung carcinomas seems to be played by TTF-1. In fact, lack of TTF-1 expression is a constant feature of poorly differentiated lung carcinomas. It has been shown that TTF-1 repressed HMGA2 expression, directly and indirectly, by inducing the expression of miR-33a, which in turn affects HMGA2 mRNA. As consequent effect, the loss of TTF-1 triggers the overexpression of HMGA2 (45, 50) (Figure 1B).

It is worth to note that there are no studies reporting HMGA expression in lung neuroendocrine tumors, or correlating HMGA expression with the major pathways underlying lung adenocarcinoma development. Then, we believe that future studies should be addressed in this direction.

## HMGA and Esophageal Carcinoma

HMGA1 and HMGA2 evaluation in esophageal carcinoma revealed interesting differences between adenocarcinoma and squamous histotypes (Table 1). In fact, increasing HMGA1 levels were observed going from low- to high-grade dysplasia (HGD) and adenocarcinoma (51). Conversely, HMGA1 mRNA and protein levels did not show any significant difference between squamous carcinoma and normal adjacent tissue. Interestingly, studies in progress in our laboratory reveal high HMGA2 expression in squamous carcinomas histotype and, conversely, its absence in normal tissue.

## HMGA and Testicular Tumors

Testicular germ cell tumors (TGCTs) represent an interesting case where the evaluation of HMGA proteins is very useful to make differential diagnosis (Table 1). Indeed, while HMGA1 was expressed in seminomas and embryonal carcinomas, by contrast, it was not detected in yolk sac carcinomas and teratomas (52). The same study reported that HMGA2 was expressed in embryonal and yolk sac carcinomas, but not in seminomas and teratomas (52).

## Epigenomic Regulation of HMGA Protein Levels

Several recent reports have highlighted the post-transcriptional repression of HMGA proteins by non-coding RNAs and, in particular, numerous miRNAs with this activity have been identified (let-7a, miR-15, miR-16, miR-26a, miR-34b, miR-196a2, miR-326, miR-432, miR-548c-3p, miR-570, miR-603) (53, 54). In most human carcinomas analyzed, quite a lot of these miRNAs were underexpressed, whereas HMGA proteins were overexpressed. In fact, low levels of miR-16 were associated with loss of differentiation, lymph node metastases, and short overall survival (Kaplan–Meyer analysis) of colorectal carcinoma patients, indicating miR-16 as an independent predictive factor of poor prognosis.

MiR-26 was downregulated in hepatocarcinoma (55) and colorectal carcinoma (56), and its loss was significantly linked to the metastatic phenotype. In addition, miR-26b was drastically downregulated in the high aggressive thyroid anaplastic carcinoma, whereas its levels did not change in the papillary and follicular histotypes, less aggressive thyroid carcinoma entities (57). A strong decrease of let-7 expression levels has been associated with an aberrant overexpression of HMGA1 and HMGA2 in several human highly malignant carcinomas (58, 59).

MicroRNAs of the miR-34b family have been found regularly underexpressed in human carcinomas and the attempt to restore their physiological levels in cancer cells currently would represent an innovative and fascinating cancer therapy (60). Intriguingly, miR-34 and HMGA1 generate an intricate regulatory loop since HMGA1 is able to negatively regulate the expression of miR-34 (Puca, unpublished observations) and p53 (61), being the latter able to induce the expression of miR-34. In this process, HMGA1 has a central role since, upon its overexpression, alters miR-34 pathway by acting directly and indirectly on it, through the repression of p53 (Figure 1C). Because of its involvement in determining the symmetric or asymmetric cell division, this pathway would play a critical role in the determination of cancer stem cell fate (61).

To render even more complicated the epigenetic regulation of HMGA, very recently, a role played by HMGA1 pseudogenes was proposed. Pseudogenes represent ancestral relatives of genes that are not any more functional, having lost the possibility to codify for proteins (62). Recently, two HMGA1 pseudogenes have been isolated (HMGA1P6 and HMGA1P7) that act by binding to miRNAs targeting HMGA1, then entrapping them, and allow the expression of functional HMGA1 gene (63). Hence, their overexpression correlates with high HMGA1 levels and malignancy grade in thyroid anaplastic, ovarian, and larynx carcinomas (63). Intriguingly, in the 3′-UTR of HMGA1, HMGA1P6, and HMGA1P7, potential binding sites for miRNAs targeting HMGA2 are also located (Figure 1D). Moreover, it is worthy to note that the 3′-UTR of HMGA2 carries as many as seven let-7 binding sites, then taking also part in the modulation of HMGA1 expression levels (47). Therefore, based on these findings, it becomes clear that not only pseudogenes, but also HMGA1 and HMGA2 themselves play a synergistical role in the control of their own expression through the miRNA decoy mechanism, leading to the establishment of extremely malignant phenotype.

Finally, the long non-coding (lnc) RNA RPSAP52 able to regulate the expression of HMGA2 has been recently identified. It has been found highly overexpressed in pituitary adenomas, where HMGA2 overexpression plays a central role in the tumorigenesis of pituitary gland, and in anaplastic thyroid carcinomas that express very high HMGA2 levels (D’Angelo, unpublished observations).

## Conclusion and Perspectives

The whole collection of published papers dealing with the expression of HMGA proteins in human malignancies clearly supports the link between HMGA overexpression and the highly malignant phenotype resulting in poor prognosis of the cancer patients. The ability to induce EMT, a crucial step during the acquisition of highly aggressive phenotype, and the ability to confer resistance to antineoplastic drugs likely account for the association of HMGA overexpression with cancer progression. Equally important appears the ability of HMGA1 to allow CSC to symmetrically divide, sustaining their stemness-like phenotype (61). In this respect, the evaluation of HMGA protein expression might represent a useful tool for the prediction of prognosis and drug response. Moreover, the recent finding that the antineoplastic drug trabectedin exerts its cytotoxic effects on carcinoma cells impairing the function of HMGA proteins (64) may suggest trabectedin treatment in patients overexpressing HMGA. In addition, further evaluation of miRNAs, lnc RNAs, and pseudogenes regulating HMGA proteins, could reinforce the importance of HMGA1 and HMGA2 as tumor markers. Currently, a stimulating challenge in diagnostic research would be the possibility to identify very little amounts of HMGA proteins directly in the blood specimens, either to make an early diagnosis or monitor the efficacy of cancer therapy. The optimization of nanotechnology-based devices is currently in progress and will allow the detection with high specificity and sensitivity of HMGA1 protein directly in the blood of CRC patients.

## Conflict of Interest Statement

The authors declare that the research was conducted in the absence of any commercial or financial relationships that could be construed as a potential conflict of interest. The Associate Editor Luigi Insabato declares no conflict of interest, and despite being affiliated to the same University as authors Pierlorenzo Pallante, Romina Sepe, Francesca Puca, and Alfredo Fusco, the review process was handled objectively.

## References

[1] Bigbee W Herberman RB Tumor markers and immunodiagnosis. In: Kufe DW Pollock RE Weichselbaum RR Bast RC Gansler TS Holland JF editors. Cancer Medicine. Hamilton, ON: BC Decker (2003). p. 209–20.

[2] Giancotti V Berlingieri MT DiFiore PP Fusco A Vecchio G Crane-Robinson C. Changes in nuclear proteins on transformation of rat epithelial thyroid cells by a murine sarcoma retrovirus. Cancer Res (1985) 45:6051–7. 2998592

[3] Giancotti V Pani B D’Andrea P Berlingieri MT Di Fiore PP Fusco A Elevated levels of a specific class of nuclear phosphoproteins in cells transformed with v-ras and v-mos oncogenes and by cotransfection with c-myc and polyoma middle T genes. EMBO J (1987) 6:1981–7. 2820715 10.1002/j.1460-2075.1987.tb02461.x PMC553586

[4] Johnson KR Lehn DA Reeves R. Alternative processing of mRNAs encoding mammalian chromosomal high-mobility-group proteins HMG-I and HMG-Y. Mol Cell Biol (1989) 9:2114–23. 2701943 10.1128/mcb.9.5.2114-2123.1989 PMC363005

[5] Nagpal S Ghosn C DiSepio D Molina Y Sutter M Klein ES Retinoid-dependent recruitment of a histone H1 displacement activity by retinoic acid receptor. J Biol Chem (22568) 274(1999):22563–8. 10.1074/jbc.274.32.22563 10428834

[6] Reeves R Nissen MS. The A.T-DNA-binding domain of mammalian high mobility group I chromosomal proteins. A novel peptide motif for recognizing DNA structure. J Biol Chem (1990) 265:8573–82. 1692833

[7] Fedele M Fusco A HMGA and cancer. Biochim Biophys Acta (2010) 1799:48–54 10.1016/j.bbagrm.2009.11.007 20123067

[8] Zhou X Benson KF Ashar HR Chada K. Mutation responsible for the mouse pygmy phenotype in the developmentally regulated factor HMGI-C. Nature (1995) 376:771–4. 10.1038/376771a0 7651535

[9] Chiappetta G Avantaggiato V Visconti R Fedele M Battista S Trapasso F High level expression of the HMGI (Y) gene during embryonic development. Oncogene (1996) 13:2439–46. 8957086

[10] Anand A Chada K. In vivo modulation of HMGIC reduces obesity. Nat Genet (2000) 24:377–80. 10.1038/74207 10742101

[11] Chieffi P Battista S Barchi M Di Agostino S Pierantoni GM Fedele M HMGA1 and HMGA2 protein expression in mouse spermatogenesis. Oncogene (2002) 21:3644–50. 10.1038/sj.onc.1205501 12032866

[12] Di Agostino S Fedele M Chieffi P Fusco A Rossi P Geremia R Phosphorylation of high-mobility group protein A2 by Nek2 kinase during the first meiotic division in mouse spermatocytes. Mol Biol Cell (2004) 15:1224–32. 10.1091/mbc.E03-09-0638 14668482 PMC363112

[13] Baldassarre G Fedele M Battista S Vecchione A Klein-Szanto AJ Santoro M Onset of natural killer cell lymphomas in transgenic mice carrying a truncated HMGI-C gene by the chronic stimulation of the IL-2 and IL-15 pathway. Proc Natl Acad Sci U S A (2001) 98:7970–5. 10.1073/pnas.141224998 11427729 PMC35452

[14] Xu Y Sumter TF Bhattacharya R Tesfaye A Fuchs EJ Wood LJ The HMG-I oncogene causes highly penetrant, aggressive lymphoid malignancy in transgenic mice and is overexpressed in human leukemia. Cancer Res (2004) 64:3371–5. 10.1158/0008-5472.CAN-04-0044 15150086

[15] Fedele M Pentimalli F Baldassarre G Battista S Klein-Szanto AJ Kenyon L Transgenic mice overexpressing the wild-type form of the HMGA1 gene develop mixed growth hormone/prolactin cell pituitary adenomas and natural killer cell lymphomas. Oncogene (2005) 24:3427–35. 10.1038/sj.onc.1208501 15735694

[16] Belton A Gabrovsky A Bae YK Reeves R Iacobuzio-Donahue C Huso DL HMGA1 induces intestinal polyposis in transgenic mice and drives tumor progression and stem cell properties in colon cancer cells. PLoS One (2012) 7:e30034. 10.1371/journal.pone.0030034 22276142 PMC3262796

[17] Fusco A Fedele M Roles of HMGA proteins in cancer. Nat Rev Cancer (2007) 7:899–910 10.1038/nrc2271 18004397

[18] Fedele M Fidanza V Battista S Pentimalli F Klein-Szanto AJ Visone R Haploinsufficiency of the Hmga1 gene causes cardiac hypertrophy and myelo-lymphoproliferative disorders in mice. Cancer Res (2006) 66:2536–43. 10.1158/0008-5472.CAN-05-1889 16510570

[19] Foti D Chiefari E Fedele M Iuliano R Brunetti L Paonessa F Lack of the architectural factor HMGA1 causes insulin resistance and diabetes in humans and mice. Nat Med (2005) 11:765–73. 10.1038/nm1254 15924147

[20] Federico A Forzati F Esposito F Arra C Palma G Barbieri A Hmga1/Hmga2 double knock-out mice display a “superpygmy” phenotype. Biol Open (2014) 3:372–8. 10.1242/bio.20146759 24728959 PMC4021359

[21] Fedele M Visone R De Martino I Troncone G Palmieri D Battista S HMGA2 induces pituitary tumorigenesis by enhancing E2F1 activity. Cancer Cell (2006) 9:459–71. 10.1016/j.ccr.2006.04.024 16766265

[22] Abe N Watanabe T Sugiyama M Uchimura H Chiappetta G Fusco A Determination of high mobility group I(Y) expression level in colorectal neoplasias: a potential diagnostic marker. Cancer Res (1999) 59:1169–74. 10096541

[23] Chiappetta G Manfioletti G Pentimalli F Abe N Di Bonito M Vento MT High mobility group HMGI(Y) protein expression in human colorectal hyperplastic and neoplastic diseases. Int J Cancer (2001) 91:147–51. 10.1002/1097-0215(200002)9999:9999<::AID-IJC1033>3.3.CO;2-M 11146437

[24] Cleynen I Huysmans C Sasazuki T Shirasawa S Van de Ven W Peeters K. Transcriptional control of the human high mobility group A1 gene: basal and oncogenic Ras-regulated expression. Cancer Res (2007) 67:4620–9. 10.1158/0008-5472.CAN-06-4325 17510387

[25] Bush BM Brock AT Deng JA Nelson RA Sumter TF. The Wnt/beta-catenin/T-cell factor 4 pathway up-regulates high-mobility group A1 expression in colon cancer. Cell Biochem Funct (2013) 31:228–36. 10.1002/cbf.2876 22961697 PMC3616152

[26] Helmke BM Markowski DN Meyer A Bullerdiek J. The expression of HMGA2 varies strongly among colon carcinomas. Anticancer Res (2012) 32:1589–93. 22593436

[27] Li Y Zhao Z Xu C Zhou Z Zhu Z You T. HMGA2 induces transcription factor Slug expression to promote epithelial-to-mesenchymal transition and contributes to colon cancer progression. Cancer Lett (2014) 355:130–40. 10.1016/j.canlet.2014.09.007 25218351

[28] Wang X Liu X Li AY Chen L Lai L Lin HH Overexpression of HMGA2 promotes metastasis and impacts survival of colorectal cancers. Clin Cancer Res (2011) 17:2570–80. 10.1158/1078-0432.CCR-10-2542 21252160 PMC3079060

[29] Cserni G Francz M Kalman E Kelemen G Komjathy DC Kovacs I Estrogen receptor negative and progesterone receptor positive breast carcinomas-how frequent are they? Pathol Oncol Res (2011) 17:663–8. 10.1007/s12253-011-9366-y 21267685

[30] Rakha EA El-Sayed ME Green AR Paish EC Powe DG Gee J Biologic and clinical characteristics of breast cancer with single hormone receptor positive phenotype. J Clin Oncol (2007) 25:4772–8. 10.1200/JCO.2007.12.2747 17876012

[31] Rhodes A Jasani B The oestrogen receptor-negative/progesterone receptor-positive breast tumour: a biological entity or a technical artefact? J Clin Pathol (2009) 62:95–6 10.1136/jcp.2008.060723 19103868

[32] Yu KD Di GH Wu J Lu JS Shen KW Liu GY Breast cancer patients with estrogen receptor-negative/progesterone receptor-positive tumors: being younger and getting less benefit from adjuvant tamoxifen treatment. J Cancer Res Clin Oncol (2008) 134:1347–54. 10.1007/s00432-008-0414-2 18488249 PMC12161716

[33] Chiappetta G Botti G Monaco M Pasquinelli R Pentimalli F Di Bonito M HMGA1 protein overexpression in human breast carcinomas: correlation with ErbB2 expression. Clin Cancer Res (2004) 10:7637–44. 10.1158/1078-0432.CCR-04-0291 15569996

[34] Mansueto G Forzati F Ferraro A Pallante P Bianco M Esposito F Identification of a new pathway for tumor progression: microRNA-181b Up-regulation and CBX7 down-regulation by HMGA1 protein. Genes Cancer (2010) 1:210–24. 10.1177/1947601910366860 21779448 PMC3092193

[35] Wend P Runke S Wend K Anchondo B Yesayan M Jardon M WNT10B/beta-catenin signalling induces HMGA2 and proliferation in metastatic triple-negative breast cancer. EMBO Mol Med (2013) 5:264–79. 10.1002/emmm.201201320 23307470 PMC3569642

[36] Jones AM Mitter R Poulsom R Gillett C Hanby AM Tomlinson IP mRNA expression profiling of phyllodes tumours of the breast: identification of genes important in the development of borderline and malignant phyllodes tumours. J Pathol (2008) 216:408–17. 10.1002/path.2439 18937276

[37] Piscuoglio S Zlobec I Pallante P Sepe R Esposito F Zimmermann A HMGA1 and HMGA2 protein expression correlates with advanced tumour grade and lymph node metastasis in pancreatic adenocarcinoma. Histopathology (2012) 60:397–404. 10.1111/j.1365-2559.2011.04121.x 22276603

[38] Hristov AC Cope L Di Cello F Reyes MD Singh M Hillion JA HMGA1 correlates with advanced tumor grade and decreased survival in pancreatic ductal adenocarcinoma. Mod Pathol (2009) 23:98–104. 10.1038/modpathol.2009.139 19820691 PMC3092591

[39] Masciullo V Baldassarre G Pentimalli F Berlingieri MT Boccia A Chiappetta G HMGA1 protein over-expression is a frequent feature of epithelial ovarian carcinomas. Carcinogenesis (2003) 24:1191–8. 10.1093/carcin/bgg075 12807722

[40] Mahajan A Liu Z Gellert L Zou X Yang G Lee P HMGA2: a biomarker significantly overexpressed in high-grade ovarian serous carcinoma. Mod Pathol (2010) 23:673–81. 10.1038/modpathol.2010.49 20228781

[41] Califano D Pignata S Losito NS Ottaiano A Greggi S De Simone V High HMGA2 expression and high body mass index negatively affect the prognosis of patients with ovarian cancer. J Cell Physiol (2014) 229:53–9. 10.1002/jcp.24416 23765903

[42] Hetland TE Holth A Kaern J Florenes VA Trope CG Davidson B. HMGA2 protein expression in ovarian serous carcinoma effusions, primary tumors, and solid metastases. Virchows Arch (2012) 460:505–13. 10.1007/s00428-012-1228-9 22476403

[43] Wu J Liu Z Shao C Gong Y Hernando E Lee P HMGA2 overexpression-induced ovarian surface epithelial transformation is mediated through regulation of EMT genes. Cancer Res (2011) 71:349–59. 10.1158/0008-5472.CAN-10-2550 21224353 PMC4434602

[44] Zhang Z Wang Q Chen F Liu J. Elevated expression of HMGA1 correlates with the malignant status and prognosis of non-small cell lung cancer. Tumour Biol (2014) 36(2):1213–9. 10.1007/s13277-014-2749-4 25344216

[45] Rice SJ Lai SC Wood LW Helsley KR Runkle EA Winslow MM MicroRNA-33a mediates the regulation of high mobility group AT-hook 2 gene (HMGA2) by thyroid transcription factor 1 (TTF-1/NKX2-1). J Biol Chem (2013) 288:16348–60. 10.1074/jbc.M113.474643 23625920 PMC3675572

[46] Kettunen E Anttila S Seppanen JK Karjalainen A Edgren H Lindstrom I Differentially expressed genes in nonsmall cell lung cancer: expression profiling of cancer-related genes in squamous cell lung cancer. Cancer Genet Cytogenet (2004) 149:98–106 10.1016/S0165-4608(03)00300-5 15036884

[47] Kumar MS Armenteros-Monterroso E East P Chakravorty P Matthews N Winslow MM HMGA2 functions as a competing endogenous RNA to promote lung cancer progression. Nature (2014) 505:212–7. 10.1038/nature12785 24305048 PMC3886898

[48] Zhang Y Ma T Yang S Xia M Xu J An H High-mobility group A1 proteins enhance the expression of the oncogenic miR-222 in lung cancer cells. Mol Cell Biochem (2011) 357:363–71. 10.1007/s11010-011-0907-1 21656127

[49] Visone R Russo L Pallante P De Martino I Ferraro A Leone V MicroRNAs (miR)-221 and miR-222, both overexpressed in human thyroid papillary carcinomas, regulate p27Kip1 protein levels and cell cycle. Endocr Relat Cancer (2007) 14:791–8. 10.1677/ERC-07-0129 17914108

[50] Winslow MM Dayton TL Verhaak RG Kim-Kiselak C Snyder EL Feldser DM Suppression of lung adenocarcinoma progression by Nkx2-1. Nature (2011) 473:101–4. 10.1038/nature09881 21471965 PMC3088778

[51] Chen X Lechago J Ertan A Ergun G Verm R Bridges M Expression of the high mobility group proteins HMGI(Y) correlates with malignant progression in Barrett’s metaplasia. Cancer Epidemiol Biomarkers Prev (2004) 13:30–3. 10.1158/1055-9965.EPI-03-0151 14744729

[52] Franco R Esposito F Fedele M Liguori G Pierantoni GM Botti G Detection of high-mobility group proteins A1 and A2 represents a valid diagnostic marker in post-pubertal testicular germ cell tumours. J Pathol (2008) 214:58–64. 10.1002/path.2249 17935122

[53] D’Angelo D Palmieri D Mussnich P Roche M Wierinckx A Raverot G Altered microRNA expression profile in human pituitary GH adenomas: down-regulation of miRNA targeting HMGA1, HMGA2, and E2F1. J Clin Endocrinol Metab (2012) 97:E1128–38. 10.1210/jc.2011-3482 22564666

[54] Palmieri D D’Angelo D Valentino T De Martino I Ferraro A Wierinckx A Downregulation of HMGA-targeting microRNAs has a critical role in human pituitary tumorigenesis. Oncogene (2012) 31:3857–65. 10.1038/onc.2011.557 22139073

[55] Ji J Shi J Budhu A Yu Z Forgues M Roessler S MicroRNA expression, survival, and response to interferon in liver cancer. N Engl J Med (2009) 361:1437–47. 10.1056/NEJMoa0901282 19812400 PMC2786938

[56] Qian J Jiang B Li M Chen J Fang M. Prognostic significance of microRNA-16 expression in human colorectal cancer. World J Surg (2013) 37:2944–9. 10.1007/s00268-013-2205-4 24045965

[57] Visone R Pallante P Vecchione A Cirombella R Ferracin M Ferraro A Specific microRNAs are downregulated in human thyroid anaplastic carcinomas. Oncogene (2007) 26:7590–5. 10.1038/sj.onc.1210564 17563749

[58] Mu G Liu H Zhou F Xu X Jiang H Wang Y Correlation of overexpression of HMGA1 and HMGA2 with poor tumor differentiation, invasion, and proliferation associated with let-7 down-regulation in retinoblastomas. Hum Pathol (2010) 41:493–502. 10.1016/j.humpath.2009.08.022 20004941

[59] Rahman MM Qian ZR Wang EL Sultana R Kudo E Nakasono M Frequent overexpression of HMGA1 and 2 in gastroenteropancreatic neuroendocrine tumours and its relationship to let-7 downregulation. Br J Cancer (2009) 100:501–10. 10.1038/sj.bjc.6604883 19156147 PMC2658538

[60] Wang R Ma J Wu Q Xia J Miele L Sarkar FH Functional role of miR-34 family in human cancer. Curr Drug Targets (2013) 14:1185–91. 10.2174/13894501113149990191 23834144

[61] Puca F Colamaio M Federico A Gemei M Tosti N Bastos AU HMGA1 silencing restores normal stem cell characteristics in colon cancer stem cells by increasing p53 levels. Oncotarget (2014) 5:3234–45. 24833610 10.18632/oncotarget.1914 PMC4102806

[62] Esposito F De Martino M Forzati F Fusco A. *HMGA1*-pseudogene overexpression contributes to cancer progression. Cell Cycle (2014) 13(23):3636–9. 10.4161/15384101.2014.974440 25483074 PMC4613653

[63] Esposito F De Martino M Petti MG Forzati F Tornincasa M Federico A HMGA1 pseudogenes as candidate proto-oncogenic competitive endogenous RNAs. Oncotarget (2014) 5:8341–54. 25268743 10.18632/oncotarget.2202 PMC4226687

[64] D’Angelo D Borbone E Palmieri D Uboldi S Esposito F Frapolli R The impairment of the high mobility group A (HMGA) protein function contributes to the anticancer activity of trabectedin. Eur J Cancer (2013) 49:1142–51. 10.1016/j.ejca.2012.10.014 23149213

[65] van Iersel MP Kelder T Pico AR Hanspers K Coort S Conklin BR Presenting and exploring biological pathways with PathVisio. BMC Bioinformatics (2008) 9:399. 10.1186/1471-2105-9-399 18817533 PMC2569944

